# Comparative Analysis of Clinical Practice Guidelines for the Pharmacological Treatment of Type 2 Diabetes Mellitus in Latin America

**DOI:** 10.1007/s11892-023-01504-4

**Published:** 2023-05-01

**Authors:** Paula Andrea Taborda Restrepo, Jorge Acosta-Reyes, Andrés Estupiñan-Bohorquez, María Alejandra Barrios-Mercado, Nestor Fabián Correa Gonzalez, Alejandra Taborda Restrepo, Noël C. Barengo, Rafael Gabriel

**Affiliations:** 1 Sapyens SAS BIC, Bogotá, Colombia; 2 Departamento de Salud Pública, Universidad del Norte, Barranquilla, Colombia; 3 Hospital Universitario San Ignacio, Bogotá, Colombia; 4 Department of Translational Medicine, Herbert Wertheim College of Medicine and Department of Health Policy and Management, Robert Stempel College of Public Health and Social Work, Florida International University, Miami, FL USA; 5 Faculty of Medicine, Riga Stradins University, Riga, Latvia; 6 International Health Department, National School of Public Health, Instituto de Salud Carlos III, Madrid, Spain, World Community for Prevention of Diabetes, Madrid, Spain

**Keywords:** Type 2 diabetes mellitus, Clinical practice guidelines, Latin America, Drug therapy

## Abstract

**Purpose of Review:**

Type 2 diabetes mellitus (T2DM) is one of the leading causes of death and disability in the world. The majority of diabetes deaths (> 80%) occur in low- and middle-income countries, which are predominant in Latin America. Therefore, the purpose of this article is to compare the clinical practice guideline (CPG) for the pharmacological management of T2DM in Latin America (LA) with international reference guidelines.

**Recent Findings:**

Several LA countries have recently developed CPGs. However, the quality of these guidelines is unknown according to the AGREE II tool and taking as reference three CPGs of international impact: American Diabetes Association (ADA), European Diabetes Association (EASD), and Latin American Diabetes Association (ALAD).

**Summary:**

Ten CPGs were selected for analysis. The ADA scored > 80% on the AGREE II domains and was selected as the main comparator. Eighty percent of LA CPGs were developed before 2018. Only one was not recommended (all domains < 60%). The CPGs in LA have good quality but are outdated. They have significant gaps compared to the reference. There is a need for improvement, as proposing updates every three years to maintain the best available clinical evidence in all guidelines.

## Introduction

Type 2 diabetes mellitus (T2DM) is a chronic noncommunicable disease with a major impact on the world's population health [1]. In addition, according to the International Diabetes Federation, approximately 537 million people worldwide live with T2DM [2•]. According to the WHO, T2DM is one of the leading causes of death and disability in the Americas; it is estimated that 83 million people in this continent live with this pathology [3•].

The control of T2DM is focused on lifestyle modification and pharmacological treatment, for which there is a wide variability of recommendations in the region, making it difficult to prescribe the optimal treatment for patients, which, as a consequence, can lead to inefficiencies and a greater economic burden for patients and health systems [4, 5].

In this context, clinical practice guidelines (CPG) are a fundamental tool for the appropriate prescription of management and medications, favoring efficient and safe prescribing with an appropriate benefit–cost ratio. Due to socioeconomic and health differences, each country has its own CPG. The CPGs play an essential role as they aim to reduce unwarranted variability in clinical practice and support decision-making by healthcare professionals [6•]. These objectives can be achieved as long as the guidelines are updated and incentives are provided for proper implementation [6•, 7].

In order to know the recommendations based on scientific evidence, appropriate to the context and available resources, CPGs are developed in each country [7]. However, there are gaps and challenges in selecting the best available evidence and the methodological quality of the available CPGs due to their variability. This may limit its use as a support for informed decision making by health professionals. In addition, they do not offer clear recommendations for patients with specific conditions, which can lead to low adherence to their suggestions and difficulty in achieving therapeutic goals [5]. Specifically, for T2DM, the quality of the guidelines in some Latin American countries is unknown.

Therefore, this study compared the CPG for the pharmacological management of T2DM in Latin America with international reference guidelines.

## Materials and Methods

### Study Design

A systematic review (SR) of T2DM CPGs developed in Latin American countries was performed. For the comparative analysis, three CPGs were selected a priori as reference guidelines: the guideline developed by the American Diabetes Association (ADA) [8••], the European Association for the Study of Diabetes (EASD) [9••] and the guideline developed by the Latin American Diabetes Association (ALAD) [10••]. Key pharmacological treatment recommendations were classified, considering the following categories of patients with T2DM that could be contemplated in the CPGs, regarding the management:

1. Pharmacological management for the elderly population
2. Populations with hypoglycemia and who have presented a risk of hypoglycemia
3. Pharmacological management of patients with diabetic nephropathy
4. Patients with risk factors and/or cardiovascular disease
5. Therapeutic failure with oral antidiabetic agents
6. Patients with therapeutic failure and HbA1c above goals
7. Patients with diabetes and obesity
8. Insulin management recommendations

### Protocol Registration

The protocol for this SR was registered with PROSPERO: CRD42022292048. This manuscript complies with the recommendations of the Preferred Reporting Items for Systematic Reviews and Meta-Analyses (PRISMA) statement [11].

### Search Strategy

For the identification of the CPGs, a search strategy was designed for MEDLINE and Embase through the Ovid platform (Appendix), followed by a snowball strategy and manual search in reference databases, exclusive databases for CPG, gray literature, on the web pages of the ministries of health and/or institutions developing CPGs or health technology assessments in Latin American countries. All the above, considering the principal terms of reference for T2DM according to the Medical Subject Heading (MeSH).

### Selection of the Clinical Practice Guidelines

CPGs that met the following inclusion criteria were selected: Evidence-based T2DM CPGs; developed by scientific societies, universities, technology assessment institutes, ministries of health, or recognized public entities; developed in the Latin American countries of Colombia, Peru, Costa Rica, Panama, Guatemala, Honduras, Ecuador, Argentina, Chile, Mexico, Brazil, and the Dominican Republic, that included treatment recommendations for T2DM. CPGs were not excluded by language or date of publication. The most recently updated versions were selected in cases where different versions of the same guideline were found.

**Fig. 1:**
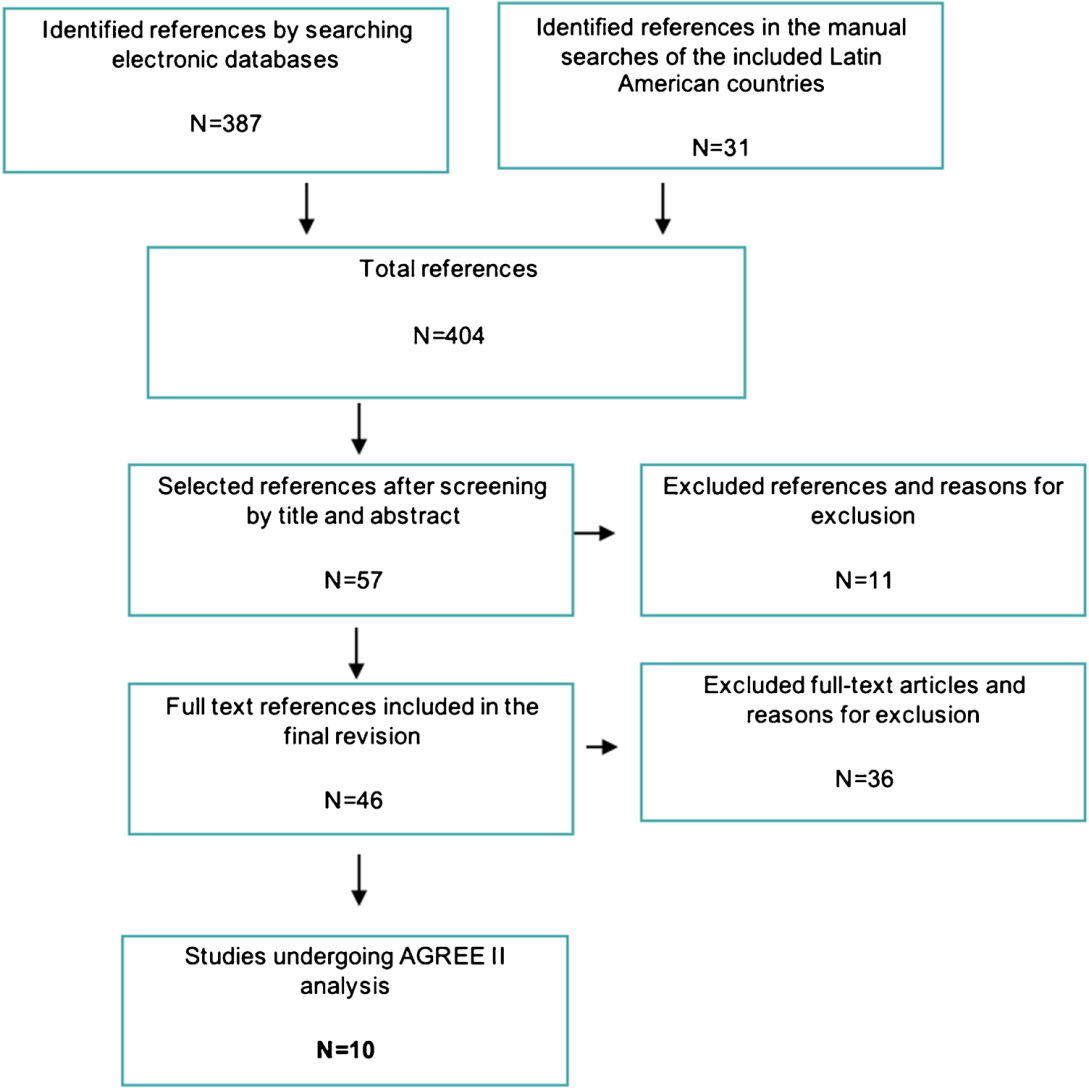
PRISMA diagram of the study: flowchart of the search, screening, and selection of CPG

For the selection of the CPGs, a format was designed in Excel version 16.54 (Microsoft Excel®Excel) that included the eligibility criteria. The process was paired and in case of disagreement a third evaluator established the consensus. The first part was developed based on the title and summary of the documents identified. The complete document was then reviewed by duplicate to verify its eligibility. The whole process is summarized in the PRISMA diagram (Fig. 1).

### Evaluation of the Methodological Quality of CPGs

The AGREE II (International Appraisal of Guidelines, Research, and Evaluation) instrument was used to evaluate the methodological quality of the CPG [7]. This instrument contains 23 key items, followed by two global scoring items. The score for each domain was calculated according to the recommendation of the instrument itself: adding all the points of the individual items of the domain and standardizing the total as a percentage over the maximum possible score for that domain. The process was paired and in case of differences of more than three points in each evaluated item, it was resolved by consensus.

Each CPG was also evaluated in a general manner considering the scoring for each domain [11].

After the evaluation of each CPG using AGREE II, pharmacological recommendations were extracted and patients with T2DM were classified according to the previously stipulated patient profiles. Subsequently, a comparison of the recommendations and the identification of gaps between the recommendations of each guideline and the reference CPG was carried out, and the aspects to be improved in each CPG were identified according to the evaluation carried out with the AGREE II tool.

Additionally, a paired evaluation of the factors supporting the recommendations related to insulin use in T2DM from each CPG was performed: (i) effectiveness of the intervention, (ii) safety/harm, (iii) evidence on patient values and preferences, (iv) use/cost considerations of the recommendation, (v) use of Grading of Recommendations Assessment, Development and Evaluation (GRADE) in the preparation of the CPG, (vi) funding, and (vii) reported conflicts of interest.

## Results

### Identification and Characteristics of the CPGs

The strategy identified 404 publications. Thirty-one papers meeting the selection criteria were found in the open search. Forty-six references that met the selection criteria were screened. Finally, 36 documents were discarded because they did not refer exclusively to the management of T2DM, or because they corresponded to duplicate references or older versions of an updated guideline. Although the Dominican Republic and Panama were prioritized a priori, no guidelines were found to have been developed in these countries. After reviewing duplicates and previous reviews, a total of 10 guidelines for the management of T2DM were selected and evaluated with the AGREE II tool (Fig. 1). The pharmacological recommendations were extracted for each Latin American guideline selected, obtaining a total of 128 recommendations. They were classified according to the patient group stipulated in the protocol.

The most recent versions of each CPG were chosen, finding one guideline published before 2010, one from 2015, one from 2016, and the remaining seven guidelines between 2017 and 2020 (Table 1). Sixty percent of the CPGs were prepared by the Ministry of Health or whoever acts in its stead, while the remaining percentage was prepared by a group of experts from different entities in each country. In Honduras, for example, international entities contributed to its preparation.

**Table 1:** Overall characteristics of the Latin American Type 2 Diabetes Guidelines identified

Country, year of publication	CPG	Authors	Year of publication	Focus
Argentina	National Clinical Practice Guideline on the prevention, diagnosis and treatment of type 2 diabetes mellitus (T2DM)	Ministry of Health (Ministerio de Salud)	2019	Prevention, diagnosis, and treatment of T2DM
Chile	Clinical Practice Guideline pharmacological treatment of Type 2 diabetes mellitus	Working group of the Clinical Practice Guideline pharmacological treatment of type 2 diabetes mellitus	2017	Pharmacological treatment
Colombia	Clinical Practice Guideline for the diagnosis, treatment, and follow-up of Type 2 diabetes mellitus in the population over 18 years old	Working Group of the Clinical Practice Guideline for the diagnosis, treatment and follow-up of Type 2 diabetes mellitus in the population over 18 years old	2016	Diagnosis, treatment, and follow-up of T2DM
Costa Rica	Guideline for the care of people with Type 2 diabetes	Costa Rican Social Security Fund (Caja costarricense de seguro social)	2007	Detection, diagnosis, treatment, education, self-management, and control
Ecuador	Clinical Practice Guideline (CPG) on Type 2 diabetes mellitus	T2DM CPG preparation group	2017	Prevention, treatment, screening, public health
Guatemala	Guideline No. 38 diabetes mellitus (update)	Guatemalan Social Security Institute (Instituto Guatemalteco de Seguridad Social)	2017	Prevention, treatment, screening, public health
Honduras	Clinical Practice Guideline for the outpatient management (promotion, prevention, diagnosis, and treatment) of adults with type 2 diabetes mellitus	Guideline preparation group: Secretary of Health from the Republic of Honduras (Secretaría de Salud de la República de Honduras)	2015	Promotion, prevention, diagnosis, and treatment
Mexico	Diagnosis and pharmacological treatment of type 2 diabetes mellitus at the first level of care CPG	Mexican Social Security Institute (Instituto Mexicano de Seguridad Social)	2018	Diagnosis and pharmacological treatment
Peru	Clinical Practice Guideline for the diagnosis, treatment, and management of type 2 diabetes mellitus at the first level of care	Ministry of Health (Ministerio de Salud) – General Direction of Strategic Interventions in Public Health (Dirección General de Intervenciones Estratégicas en Salud Pública)	2016	Treatment and control
Brazil	Clinical Protocol and Therapeutic Guidelines for type 2 diabetes mellitus	Ministry of Health (Ministerio de salud), Conitec	2020	T2DM therapy

### Evaluation of the Quality of the CPGs of the Countries

The median percentage of CPG evaluation by the AGREE II instrument (Table 2) ranged from 29 to 96%. It is important to mention that for CPGs that exceed a median evaluation percentage of 60%, their features were all above 80% in their weighted percentages of the domains (Table 3).

**Table 2:** Domain scores and general evaluations of diabetes guidelines considered as reference guidelines and of the selected countries, according to AGREE II

Country, year of publication	Scope and objective	Participation of stakeholders	Rigor in preparation	Clarity of presentation	Applicability	Editorial independence	Global score of the guideline	Global evaluation of the guideline
ALAD, 2019	39	17	25	67	10	29	3	Not recommended
ADA, 2021	89	92	85	100	100	96	6.5	Strongly recommended
EASD, 2021	64	22	51	89	2	96	3	Recommended with modifications
Argentina, 2019	97	100	90	92	73	100	6	Strongly recommended
Brazil, 2020	78	86	81	81	67	92	6	Strongly recommended
Chile, 2017	94	39	42	83	6	38	3.5	Recommended with modifications
Colombia, 2016	97	100	89	67	96	100	7	Strongly recommended
Costa Rica, 2007	58	61	50	78	42	25	3.5	Recommended with modifications
Ecuador, 2017	100	97	90	92	56	100	6	Strongly recommended
Guatemala, 2017	78	53	44	69	21	92	4	Recommended with modifications
Honduras, 2015	94	100	95	94	92	92	7	Strongly recommended
Mexico, 2018	97	81	82	86	81	92	6	Strongly recommended
Peru, 2016	44	14	14	56	31	0	2.5	Not recommended
Mean score for each domain	83.7	73.1	67.7	79.8	56.5	73.1		
Median score for each domain	94	83.5	81.5	82	61.5	92

**Table 3:** Descriptive statistics of the AGREE II score obtained by each CPG

CPG	Min**	Max**	Median***	P25***	P75***
Argentina	73%	100%	92%	90.5%	98.5%
Chile	6%	94%	42%	38.5%	67%
Colombia	67%	100%	96%	96%	98.5%
Costa Rica	25%	78%	55%	46%	59.5%
Ecuador	56%	100%	92%	89%	98.5%
Guatemala	21%	92%	57%	48.5%	73.5%
Honduras	92%	100%	94%	93%	95%
Mexico	81%	97%	86%	81.5%	89%
Peru	0%	56%	29%	14%	37.5%
Brazil	67%	92%	81%	79%	83.5%

These scores were based on the average of the AGREE-II evaluations made by four reviewers

**Minimum and maximum score in a domain of AGREE-II for each country

***Median score, 25th percentile and 75th percentile of AGREE II domains for each country

#### Domain 1. Scope and Objective

This domain refers to the general purpose of the guideline, the specific health aspects, and the target population. The mean of the evaluation was 81% (range 39–100%). In this domain, 6 CPGs scored over 80% (Argentina-Chile-Colombia-Ecuador-Honduras-Mexico).

#### Domain 2. Stakeholder participation

This domain refers to the degree to which the guideline has been developed by those involved in the preparation and it represents the point of view of users. *Stakeholders are all people who contributed to the preparation of the guideline, whether from a methodological (epidemiologists), clinical (all health personnel who see patients with diabetes), consumer (patient), economic ambit among others.* The mean for evaluation was 73% (IQR 14–100). In this domain, six CPGs scored over 80% (Argentina-Brazil-Colombia-Ecuador-Honduras-Mexico).

#### Domain 3. Rigor in Preparation

This domain refers to the process used to gather and synthesize evidence, the methods used to formulate recommendations and to update them. The mean of the evaluation was 68% (range 14–95%). In this domain, six CPGs scored over 80% (Argentina-Brazil-Colombia-Ecuador-Honduras-Mexico).

#### Domain 4. Clarity of presentation

This domain refers to the language, structure, and format of the guideline. The mean evaluation was 80% (range 56–94%). In this domain, six CPGs scored over 80% (Argentina-Brazil-Chile-Ecuador-Honduras-Mexico).

#### Domain 5. Applicability

This domain refers to the possible barriers and facilitating factors for its implementation, strategies to improve its adoption and the implications of the application of the guideline on resources. The mean evaluation was 57% (range 6–96%). In this domain, four CPGs scored over 80% (Argentina-Colombia-Honduras-Mexico).

#### Domain 6. Editorial independence

This domain is related to the formulation of recommendations not being biased by conflicts of interest. The mean of the evaluation was 73% (range 0–100%). In this domain, seven CPGs scored over 80% (Argentina-Brazil-Chile-Colombia-Ecuador-Guatemala-Honduras-Mexico).

#### Global Evaluation of Guidelines

In the global evaluation of the CPGs, which contemplates the six domains evaluated, six guidelines were classified as strongly recommended (Argentina, Brazil, Colombia, Ecuador, Honduras, Mexico). Three CPGs are recommended with modifications (Chile, Costa Rica, Guatemala). Only one CPG is not recommended (Peru), as none of the domains scored > 60%.

In the overall score of the guideline, three CPGs (Costa Rica, Guatemala, and Peru) had a score of < 4 points (2, 4, and 9).

#### General Recommendations and by Country According to the Improvement Needs of Each of the Guidelines

In the case of the Honduras CPG, of 67 treatment recommendations proposed by the ADA, 15 recommendations coincided and only one differed. For the Mexican CPG, 19 treatment recommendations were found, three of which coincide with the ADA recommendations.

The Honduran CPG is the one with the highest methodological quality profile developed in Latin America, where the general objectives of the guidelines are specifically described. There is stakeholder participation, rigor in its preparation, clarity in its presentation, applicability in its context, and editorial independence.

The CPG developed in Brazil could be improved in the description of the general objectives and stakeholder participation. Although the rigor in its preparation is good, it does not consider procedures for its updating, which detracts from its quality. Regarding to clarity of presentation, the key recommendations could be better highlighted to make them more identifiable. Improving their barriers and facilitators for implementation is a key factor, as well as are the tools on how the recommendations can be put into practice. This guideline has the necessary editorial independence.

The Peruvian CPG is the least methodologically rigorous of those developed in LA, and its improvement is essential in all respects, including the description of the general objectives, stakeholder participation, the rigor of its preparation, the clarity of its presentation, its applicability in its context and its editorial independence.

In the Mexican CPG, the general objectives of the guideline are specifically described, there is participation of stakeholders which could be complemented with a larger group of professionals and include more perspectives of the target population, a greater description of the population, the rigor in its preparation is satisfactory, as well as the clarity of its presentation. It is important to provide a greater description of barriers and facilitators to increase applicability in its context, and its editorial independence is highlighted.

### Comparison of reference CPG recommendations and country recommendations

The Latin American guidelines were compared, and it was found that none of the guidelines evaluated consider the elderly population in their treatment recommendations, unlike the reference guideline that contemplates differentiation for each of the defined profiles. Additionally, only the Honduras guideline has a recommendation on patients with T2DM who have renal complications. Three guidelines (Colombia-Honduras-Costa Rica) consider pharmacological therapy in patients with overweight; six guidelines (Honduras-Mexico-Peru-Ecuador-Costa Rica-Argentina) made recommendations based on the presence of cardiovascular disease or risk in the patients with T2DM. However, only the Argentine guideline recommends the use of an SGLT2 inhibitor in patients with established cardiovascular disease. Four guidelines (Ecuador-Peru-Costa Rica-Honduras) recommend the use of aspirin in all patients with coronary artery disease or cardiovascular risk who present T2DM. The Honduras guideline is the only one that considers patients with dyslipidemia and T2DM. Only four guidelines (Mexico-Colombia-Argentina-Honduras) made recommendations based on the risk of hypoglycemia.

Most of the guidelines (Colombia-Chile-Mexico-Brazil-Ecuador-Argentina-Honduras-Costa Rica) made recommendations based on the inadequate glycemic control of patients despite having received previous oral antidiabetic therapy. All of them recommend the use of triple therapy in patients with adequate glycemic control. It is noteworthy that Ecuador is the only guideline that recommends the use of glibenclamide associated to metformin in the event that glycemic control is not achieved. Only three guidelines (Colombia-Ecuador-Mexico) gave recommendations for patients who persist with elevated HbA1 levels despite prior treatment with oral hypoglycemic agents. Colombia and Mexico recommend combination therapy with a DPP-4 or SGLT-2 inhibitor in this patient profile. Seven guidelines (Honduras-Ecuador-Chile-Argentina-Costa Rica-Mexico-Guatemala) characterized the recommendations according to insulin therapy. All guidelines except the Honduran guideline recommend initiating therapy with NPH insulin (intermediate insulin) compared to insulin analogs. The Honduran guideline recommends starting with slow-acting insulins instead of NPH since they have been shown to be effective in reducing the risk of symptomatic nocturnal hypoglycemia.

In general, gaps are found in medication profiles and uses. Only seven recommendations for the use of insulin were found in all the CPGs consulted in six countries: Argentina, Brazil, Colombia, Ecuador, Honduras, and Mexico. Except for the Ecuadorian CPG, all the others considered the use of GRADE for the recommendation; the strength that endorsed the recommendation for the use of insulins was heterogeneous with no influence of possible conflicts of interest in all the recommendations given (Table 4).

**Table 4:** Recommendations alluding to the use of insulin in the CPGs consulted

CPG	Key recommendation	Strength of recommendation	Use of GRADE	Conflicts of interest	CPG Financing
Argentina	In patients with T2DM who initiate insulin treatment and present an increased risk of hypoglycemia, it is suggested to consider the use of long-acting insulin analogs (levemir, glargine 100 U/ml, glargine 300 U/ml or degludec), since they are similar to NPH insulin for metabolic control but present fewer cases of nocturnal hypoglycemia	Conditional, moderate quality of evidence	Yes	There were some members who declared conflicts of interest considered to have no influence on the final recommendation	Ministry of Health (Ministerio de Salud de la Nación)
Brazil	The use of the following drugs is suggested in the need to intensify hypoglycemic treatment of patients with T2DM: sulfonylureas, NPH insulin, SGLT2 and GLP-1, instead of acarbose, methyglinides, DDP4, TZD	Moderate to very low level of evidence, weak recommendation	Yes	There were some members who declared conflicts of interest considered to have no influence on the final recommendation	Ministry of Health (Ministerio de Salud)
Brazil	SGLT-2 is suggested instead of sulfonylureas or insulin for intensification in patients with T2DM	Moderate to very low level of evidence, weak recommendation	Yes	There were some members who declared conflicts of interest considered to have no influence on the final recommendation	Ministry of Health (Ministerio de Salud)
Colombia	The addition of basal insulin as a third antidiabetic medication is suggested for patients who have fail to reach their HbA1c goal with the combination of two drugs and who are not obese (BMI < 30)	Weak recommendation in favor	Yes	There were some members who declared conflicts of interest considered to have no influence on the final recommendation	Ministry of Science (Minciencias)
Ecuador	Insulin therapy should be initiated if therapeutic HbA1c objectives are not achieved after 3 months of maintaining 2 oral antidiabetics in combination at their maximum dose, or for the management of acute decompensation. Intermediate-acting insulin (isophane or NPH insulin) is recommended, at a starting subcutaneous dose of 10 IU/day, or 0.10–0.30 IU/kg/day, preferably with nocturnal onset. (153–156). The dose should be titrated progressively until therapeutic goals are achieved, maintaining periodic controls. This will vary by patient and will be decided on an individualized basis by the clinician or specialist in endocrinology, diabetes and/or insulin-trained physicians	Evidence from at least one randomized controlled clinical trial, recommendation extrapolated from the evidence (E- Ib R- B)	No	All members declared no conflicts of interest	Ecuador’s Ministry of Public Health (Ministerio de Salud Pública del Ecuador)
Honduras	The use of insulin glargine or insulin detemir instead of NPH insulin is recommended in patients with T2DM, since it appears to be effective in reducing the risk of symptomatic and nocturnal hypoglycemia, although it has not shown a beneficial effect on mortality, morbidity, quality of life or cost outcomes	Weak recommendation in favor of its use	Yes	All members declared no conflicts of interest	PAHO/WHO—Secretaría de Salud de la República de Honduras
Mexico	It is suggested that basal insulin should be added as a third drug in those adult patients with T2DM who have not achieved the HbA1c control goals or have lost it and do not suffer from obesity	A NICE (Gross J, 2011)	Yes	All members declared no conflicts of interest	Mexican Social Security Institute (Instituto mexicano del seguro social)

Regarding patient values and preferences, no information was obtained for most of the countries. Three countries reported a benefit/cost analysis that supports the recommendation of the use of some drugs, where two of them considered an increase in costs in the treatment with GLP-1 analogs justified by the benefit of the patients with this medication and in the cases in which the use of insulin analogs is not feasible. The CPG from a third country indicated no evidence of a beneficial effect of long-acting analogs on the mortality, morbidity, quality of life, or costs outcomes. Only two CPGs were found that considered aspects of feasibility, acceptability, and equity that support the recommendation. All the countries that gave recommendations regarding the use of insulins considered education for their use and/or the detection of hypoglycemia (Table 5).

**Table 5:** Considerations regarding the use of insulin in the CPGs consulted

CPG	Patient values and preferences supporting the recommendation	Benefit/cost analysis supporting the recommendation	Summary of feasibility, acceptability and equity aspects supporting the recommendation	Insulin use education and/or hypoglycemia screening
Argentina	The medication chosen by the treating physician, based on the individualization of the cases (including risk of hypoglycemia and body weight) will be accepted by the patient, considering that it will be offered in a safe and controlled manner, with periodic monitoring and evaluation of therapeutic goals by the treating health professional	The use of GLP-1 analogs may result in increased treatment costs but should be offered in cases where the use of insulin analogs is not feasible	No information	Yes
Brazil	Although NPH is preferred for most patients with T2DM who require insulin, in this subpopulation of patients at increased risk of hypoglycemia, the benefit in absolute numbers would be greater, so the use of slow-acting insulin analogs is prioritized to reduce nocturnal hypoglycemia even though they have not been shown to be more effective for glycemic control or to reduce severe hypoglycemia	Regarding costs, it was considered that although the incremental cost of using slow-acting insulin analogs vs NPH is considerable, the net benefit in these patients justifies the use of resources	It was considered that most patients would want to receive the intervention	Yes
Colombia	No information	No information	No information	Yes
Ecuador	No information	There is no evidence of a beneficial effect of long-acting analogs on mortality, morbidity, quality of life or cost outcomes	There is no evidence of a beneficial effect of long-acting analogs on mortality, morbidity, quality of life or cost outcomes	Yes
Honduras	No information	No information	No information	Yes
Mexico	No information	No information	No information	Yes

## Discussion

When CPGs are prepared in a rigorous manner, they ensure a quality that allows the extrapolation of medical knowledge into useful recommendations for daily clinical practice, which has a direct impact on patient care and has been associated with a positive impact on patient care [12, 13]. When recommendations are written without methodological rigor, trust in CPGs among clinicians is questioned and adherence to treatment is compromised. Several studies point out that the adequate quality of a guideline is what guarantees an adequate impact on health [14], while clinical and methodological reviews have documented the great variability in the quality of CPGs developed around the world [14–17].

In this SR of T2DM CPGs in Latin American countries, the quality of the guidelines evaluated by the AGREE II instrument was found to be good in general. Six out of ten CPGs identified were classified as strongly recommended, and only one CPG was not recommended due to low quality. Domain ratings were high for strongly recommended CPGs. Rigor in the elaboration domain, which has traditionally been reported as one of the most important in the preparation of CPGs, was rated above 80% in these guidelines (a high score). On the other hand, in those recommended with modifications or not recommended, the percentage of the domain rating was not above 50% (moderate or low rating). This shows an important concordance between the rigor in the elaboration and the overall quality of the CPG.

The AGREE II instrument domains “scope and objective” and “clarity of presentation” were the highest rated, this finding is similar to what was found in the evaluation of other CPGs worldwide [18, 19]. On the other hand, the lowest rated domain was “applicability”; there were three CPGs that were categorized as low (< 40%), contrasting with the expected local focus that the identified CPGs should have. It is common to find low scores in this domain in other guidelines, but the local focus reported by each of the CPGs identified shows almost no concern for the applicability of the recommendations in the target population; few CPGs have mentioned the identification of key factors for the applicability of the guidelines or plans for implementation and auditing. The need to increase training and resources is essential so that this domain is addressed more strongly in the updates of the CPGs identified with low scores.

Traditionally, it has been thought that high-quality CPG preparation processes are centered in European and North American countries. A SLR of CPG on non-insulin therapy for diabetes developed by Lam et al. [20•] found that most of the evaluated guidelines showed a wide variation in quality. However, our results on the good quality of 6 T2DM CPGs in Latin American countries demonstrate an adequate preparation and progress of the CPG process. Although this is not constant for all domains or countries, there are important regional examples that show this progress. In 2010, the national CPG project was launch by the Ministry of Health in Colombia, under the administrative and financial coordination of the Administrative Department of Science, Technology, and Innovation; Colciencias, now called Minciencias, which led to the preparation of more than 58 high quality guidelines in the country, and promoted the training of many researchers and clinical epidemiologists, as well as the development of institutions for the preparation of CPGs, such as the Alianza Centro Nacional de Investigación en Evidencia y Tecnologías en Salud (CINETS) in 2009 and the Instituto de Evaluación Tecnológica en Salud (IETS) in 2011. Similar experiences have been reported in Argentina and Mexico.

However, our results also showed the other side of the coin. The only CPG not recommended among the selected countries was the CPG from Peru, which obtained very low scores in all domains, where no score was above 60%. A study carried out in Peru that evaluated the quality of 31 CPGs found low scores in the 6 domains of AGREE II, with the lowest average scores being methodological rigor (6%) and applicability (8%). The authors concluded that there is a growing production of CPGs, but of low quality and not recommended for use [21]. This indicates the need to further promote the training and adequate preparation of CPGs in all Latin American countries.

When possibilities for T2DM control with oral antidiabetic medication and lifestyle changes have been exhausted the ADA reference indicates that patients with T2DM may benefit additionally from insulin therapy, where long-acting basal analogs demonstrate greater reduction in the risk of hypoglycemia compared to NPH insulin [8••]. It is noteworthy that in the reference documents for Latin America, of the ten CPGs found, only the use of insulin was considered in six countries with heterogeneous recommendations with moderate to very low quality of evidence in general.

Finally, five key phases in the process of translating research into practice and policy have been proposed [22, 23]. Even more relevant, the third phase of the knowledge integration process includes research designed to increase the acceptance and implementation of evidence-based recommendations such as clinical guidelines in practice, while the last phase of translational research involves the evaluation of the effectiveness and cost-effectiveness of such interventions in the "real world" and in diverse populations [24]. Therefore, future studies can also assess how well clinical practice guidelines have been implemented in different LA populations.

There is a time limitation which implies that during the development of this study, new CPGs might have been prepared and have not been included in this search. Likewise, a spatial limitation is also considered due to the selection of countries for convenience, making it impossible to extrapolate results to other contexts.

A strength of this SLR is the systematic search in the main databases of reference in the world and the search in each one of the countries of interest. This search allowed for the identification of regional CPGs that have not been recognized outside their countries of preparation, nor traditionally critically evaluated with the AGREE II instrument. The regional focus also allows for a better understanding of the reality of progress in the preparation of CPGs at the local level. Likewise, the results of this study and the comparability between the CPGs are limited to the preparation and updating such guidelines by each country, where some contain more current recommendations than others.

## Conclusions

In conclusion, after developing the comparative analysis of the current CPGs identified in Latin America with the ADA reference guidelines, multiple information gaps have been found regarding to the recommendations according to the patient profile and the pharmacological management of T2DM, especially in insulin treatment. It is worth highlighting that all the guidelines that proposed insulin therapy considered the importance of education in its use and/or the detection of hypoglycemia. A call is made for CPGs on T2DM to have continuous updates for all of their recommendations accompanied by a cost-effectiveness analysis component that supports the inclusion of new therapies suggested in their contexts.

## Data Availability

Not applicable

## Appendix. Search strategy

### MEDLINE/OVID search strategy

1. Exp clinical pathway/
2. Exp clinical protocol/
3. Exp consensus/
4. Exp consensus development conference/
5. Exp consensus development conferences as topic/
6. Critical pathways/
7. Exp guideline/
8. Guidelines as topic/
9. Exp practice guideline/
10. Practice guidelines as topic/
11. Health planning guidelines/
12. (Guideline or practice guideline or consensus development conference or consensus development conference, NIH).pt.
13. (Position statement* or policy statement* or practice parameter* or best practice*).ti,ab,kf,kw.
14. (Standards or guideline or guidelines).ti,kf,kw.
15. (Standards or guideline or guidelines).ti,kf,kw.
16. ((practice or treatment* or clinical) adj guideline*).ab.
17. (CPGs or CPGs).ti.
18. Consensus*.ti,kf,kw.
19. consensus*.ab./freq=2
20. ((Critical or clinical or practice) adj2 (path or paths or pathway or pathways or protocol*)).ti,ab,kf,kw.
21. recommendat*.ti,kf,kw.
22. (care adj2 (standard or path or paths or pathway or pathways or map or maps or plan or plans)).ti,ab,kf,kw.
23. (Algorithm* adj2 (screening or examination or test or test or test or testing or assessment* or diagnosis or diagnoses or diagnosed or diagnosing)).ti,ab,kf,kw.
24. (Algorithm* adj2 (pharmacotherap* or chemotherap* or chemotreatment* or therap* or treatment* or intervention*)).ti,ab,kf,kw.
25. or/1–24
26. Exp diabetes mellitus, Type 2/or diabetes.mp.26 and 25
27. Limit 26 to yr =”2000-Current”
28. Limit 27 to humans

### EMBASE/OVID Search strategy

1. Exp clinical pathway/
2. Exp clinical protocol/
3. Exp consensus/
4. Exp consensus development conference/
5. Exp consensus development conferences as topic/
6. Exp critical pathways/
7. Exp practice guideline/
8. Exp practice guidelines as topic/
9. (Position statement$ or policy statement$ or practice parameter$ or best practice$).ti,ab,kw.
10. ((Practice or treatment$ or clinical) adj standards).tw,kw.
11. ((Practice or treatment$ or clinical) adj guideline$).tw,kw.
12. CPG$.tw.
13. Consensus.ti.
14. ((Critical or clinical or practice) adj2 (path or paths or pathway or pathways or protocol$)).tw,kw.
15. or/1-14
16. Exp diabetes mellitus, Type 2/ or diabetes.mp.
17. 15 and 25
18. Limit 17 to yr = “2000-current”
19. Limit 18 to humans
